# Metabolomic differentiation of benign vs malignant pulmonary nodules with high specificity via high-resolution mass spectrometry analysis of patient sera

**DOI:** 10.1038/s41467-023-37875-1

**Published:** 2023-04-24

**Authors:** Yao Yao, Xueping Wang, Jian Guan, Chuanbo Xie, Hui Zhang, Jing Yang, Yao Luo, Lili Chen, Mingyue Zhao, Bitao Huo, Tiantian Yu, Wenhua Lu, Qiao Liu, Hongli Du, Yuying Liu, Peng Huang, Tiangang Luan, Wanli Liu, Yumin Hu

**Affiliations:** 1 Sate Key Laboratory of Biocontrol, School of Life Sciences, Sun Yat-sen University, Guangzhou, 510275 Guangdong China; 2 Department of Clinical Laboratory, State Key Laboratory of Oncology in South China, Sun Yat-sen University Cancer Center, Guangzhou, 510060 Guangdong China; 3 Department of Radiology, The First Affiliated Hospital of Sun Yat-sen University, Guangzhou, 510080 Guangdong China; 4 State Key Laboratory of Oncology in South China, Collaborative Innovation Center for Cancer Medicine, Sun Yat-sen University Cancer Center, Guangzhou, 510060 Guangdong China; 5 Metabolomics Research Center, Zhongshan School of Medicine, Sun Yat-sen University, Guangzhou, 510080 Guangdong China; 6 Department of Pathology, The First Affiliated Hospital of Sun Yat-sen University, Guangzhou, 510080 Guangdong China; 7 School of Biology and Biological Engineering, South China University of Technology, Guangzhou, 510006 Guangdong China; 8 Institute of Environmental and Ecological Engineering, Guangdong University of Technology, Guangzhou, 510006 Guangdong China

**Keywords:** Cancer, Biomarkers

## Abstract

Differential diagnosis of pulmonary nodules detected by computed tomography (CT) remains a challenge in clinical practice. Here, we characterize the global metabolomes of 480 serum samples including healthy controls, benign pulmonary nodules, and stage I lung adenocarcinoma. The adenocarcinoma demonstrates a distinct metabolomic signature, whereas benign nodules and healthy controls share major similarities in metabolomic profiles. A panel of 27 metabolites is identified in the discovery cohort (*n* = 306) to distinguish between benign and malignant nodules. The discriminant model achieves an AUC of 0.915 and 0.945 in the internal validation (*n* = 104) and external validation cohort (*n* = 111), respectively. Pathway analysis reveals elevation in glycolytic metabolites associated with decreased tryptophan in serum of lung adenocarcinoma vs benign nodules and healthy controls, and demonstrates that uptake of tryptophan promotes glycolysis in lung cancer cells. Our study highlights the value of the serum metabolite biomarkers in risk assessment of pulmonary nodules detected by CT screening.

## Abstract (web summary)

Detection of lung adenocarcinoma through serum sampling could be an alternative to CT scanning. Here, the authors use global metabolomics to create a 27 metabolite signature, which showed accuracy in detection in an external validation cohort.

## Introduction

Early diagnosis is vital to improve the survival rate of cancer patients. Results from the American National Lung Cancer Screening Trial (NLST) and the European NELSON trial both demonstrated that screening with low-dose computed tomography (LDCT) significantly reduces lung cancer mortality in high-risk individuals^1–3^. After the widespread use of LDCT for lung cancer screening, incidental radiographic findings of asymptomatic pulmonary nodules have continued to rise^4^. A lung nodule is defined as a focal opacity measuring up to 3 cm in diameter^5^. We are facing challenges in assessing the probability of malignancy and managing a large number of pulmonary nodules incidentally found by LDCT. The limitation of CT may cause frequent follow-up examinations and false-positive findings leading to unnecessary interventions and overtreatments^6^. Therefore, there is a need for developing reliable and convenient biomarkers to correctly identify lung cancer at the early stage and distinguish a majority of benign nodules at initial discovery^7^.

Comprehensive molecular analysis of the blood (serum, plasma, peripheral blood mononuclear cells), including genomics, proteomics, or DNA methylation^8–10^, has attracted growing interest in discovering biomarkers for lung cancer diagnosis. Meanwhile, the metabolomic approach measures the cellular end products influenced by both endogenous and exogenous activities and hence has been applied to predict disease onset and outcome. Liquid chromatography-tandem mass spectrometry (LC-MS) is a widely used approach for metabolomic study because of its high sensitivity and large dynamic range to cover metabolites with various physicochemical properties^11–13^. Although the global metabolomic profiling of plasma/serum has been applied to identify biomarkers associated with diagnosis^14–17^ and therapeutic efficacy in lung cancer^18^, the serum metabolite classifier to discriminate between benign and malignant lung nodules remains to be investigated in a large-cohort study.

Adenocarcinoma and squamous cell carcinoma are two major subtypes of non-small cell lung cancer (NSCLC). It has been shown that adenocarcinoma was the most frequent histology of lung cancer found in various CT screening trials^1,19–21^. In the current study, we perform metabolomic analysis with ultra-performance liquid chromatography- high resolution mass spectrometry (UPLC-HRMS) on a total of 695 serum samples including healthy controls, benign lung nodules and stage I lung adenocarcinoma ≤3 cm detected by CT screening. We identify a panel of serum metabolites that distinguish lung adenocarcinoma from benign nodules and healthy controls. Pathway enrichment analysis shows that aberrant tryptophan and glucose metabolism are common alterations in lung adenocarcinoma compared with benign nodules and healthy controls. Finally, we establish and validate a serum metabolic classifier with high specificity and sensitivity in differentiating malignant and benign pulmonary nodules detected by LDCT, which may facilitate differential diagnosis and risk assessment at the early stage.

## Results

### Study populations and patient characteristics

In the current study, serum samples matched according to gender and age, were collected retrospectively from 174 healthy controls, 292 subjects with benign pulmonary nodules, and 229 patients with stage I lung adenocarcinoma. The demographic characteristics of the 695 subjects are shown in Supplementary Table 1.

**Fig. 1:**
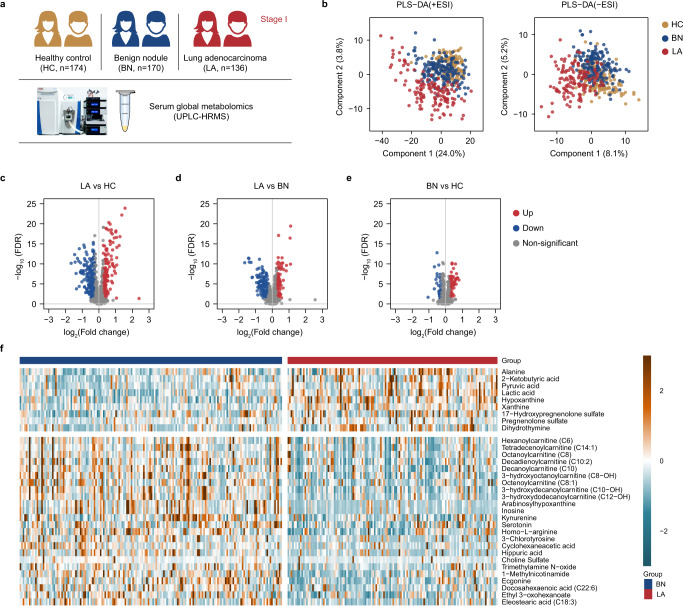
Significant perturbation in the serum metabolome of lung adenocarcinoma compared with healthy controls and benign nodules. **a** The study population of serum global metabolomic analysis by ultra-performance liquid chromatography-high resolution mass spectrometry (UPLC-HRMS) in the discovery cohort. **b** The partial least squares discrimination analysis (PLS-DA) of the global metabolomes of 480 serum samples in the discovery cohort including heathy controls (HC, *n* = 174), benign nodules (BN, *n* = 170), and stage I lung adenocarcinoma (LA, *n* = 136). +ESI, positive electrospray ionization mode; −ESI, negative electrospray ionization mode. **c–e** Significant differentially abundant metabolites between two given groups (Two-sided Wilcoxon rank tests with the *p* value adjusted by false discovery rate, FDR < 0.05) are shown in red (fold change > 1.2) and blue (fold change < 0.83) in the volcano plot. **f** A hierarchical clustering heat map showing significantly differential abundance of annotated metabolites between LA and BN. Source data are provided as a Source Data file.

As shown in Fig. 1a, a total of 480 serum samples collected from Sun Yat-sen University Cancer Center, including 174 healthy controls (HC), 170 benign nodules (BN) and 136 stage I lung adenocarcinoma (LA) were used as the discovery cohort for untargeted metabolomic analysis by ultra-performance liquid chromatography- high resolution mass spectrometer (UPLC–HRMS). As the study workflow shown in Supplementary Fig. 1, differential metabolites between LA and HC, LA and BN were identified to build the classification model and further studied for differential pathway analysis. 104 samples collected from Sun Yat-sen University Cancer Center and 111 samples collected from another two hospitals were assigned for internal and external validation, respectively.

### Serum metabolomic profiling of healthy controls, benign pulmonary nodules and lung adenocarcinoma

UPLC-HRMS analysis was conducted to profile the global serum metabolomes of 174 HC, 170 BN and 136 LA in the discovery cohort. We first showed that quality control (QC) samples were closely clustered in the center of the unsupervised principal component analysis (PCA) model, validating the performance stability of the current study (Supplementary Fig. 2).

As demonstrated by the partial least squares discrimination analysis (PLS-DA) in Fig. 1b, we found a clear profile separation between LA and BN, LA and HC, in both positive (+ESI) and negative (−ESI) electrospray ionization mode. However, no significant discrimination was found between BN and HC in either +ESI or −ESI mode.

We found 382 differential features in LA vs HC, and 231 in LA vs BN, respectively, whereas 95 in BN vs HC (Wilcoxon rank test, FDR < 0.05 and fold change >1.2 or <0.83) (Fig. 1c–e). Peaks were further annotated (Supplementary Data 3), based on database (mzCloud/HMDB/Chemspider library) searching by *m*/*z* value, retention time, and fragmentation mass spectrum (details described in Methods)^22^. 33 and 38 annotated metabolites with significantly differential abundance were finally identified for LA vs BN (Fig. 1f and Supplementary Table 2) and LA vs HC (Supplementary Fig. 3 and Supplementary Table 2), respectively. In contrast, only 3 metabolites were identified with significant differential abundance in BN vs HC (Supplementary Table 2), which is in agreement with the overlap between BN and HC in PLS-DA. These differential metabolites covered a wide range of biochemicals (Supplementary Fig. 4). Taken together, these results demonstrate a substantial alteration in the serum metabolome that reflects the malignant transformation in the early-state lung cancer compared with either benign pulmonary nodules or healthy subjects. Meanwhile, the similarity in serum metabolomes of BN and HC indicates that benign pulmonary nodules may share a number of common biological features with healthy subjects. Considering that mutations in the epidermal growth factor receptor (EGFR) gene are commonly found in the subtype of lung adenocarcinoma^23^, we sought to determine the impact of the driver mutation on serum metabolomes. We then analyzed the global metabolomic profiles of 72 cases with available EGFR status in the lung adenocarcinoma group. Interestingly, we found comparable profiles between EGFR-mutant (*n* = 41) and EGFR-wild type (*n* = 31) patients in the PCA analysis (Supplementary Fig. 5a). However, we identified 7 metabolites with significantly altered abundance in EGFR-mutant compared with EGFR-wild type (*t*-test, *p* < 0.05 and fold change >1.2 or <0.83) (Supplementary Fig. 5b) patients. The majority of these metabolites (5 out of 7) were acylcarnitines that play important roles in the fatty acid oxidation pathway.

### Establishing, tuning and validating the classification model with differential serum metabolites

**Fig. 2:**
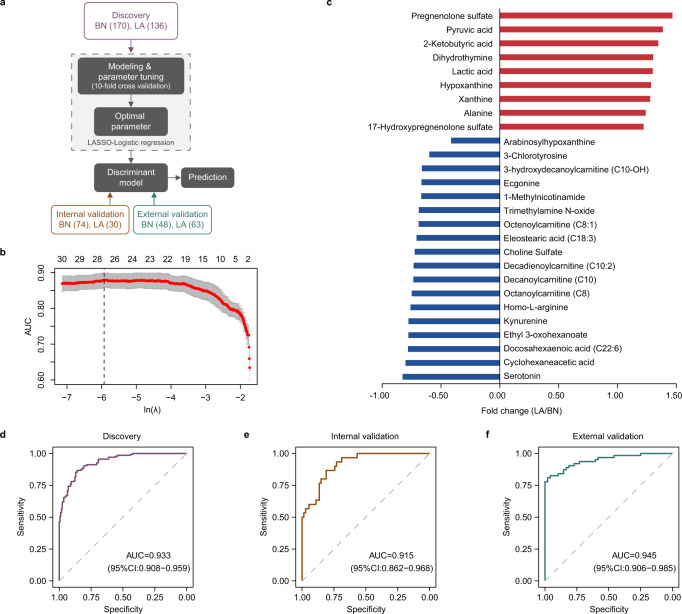
Construction and validation of the serum metabolic classifier for discriminating between benign and malignant pulmonary nodules. **a** The establishment workflow of the pulmonary nodule classifier, including selection of an optimal serum metabolite panel in the discover set by tenfold cross validation using binary logistic regression model, and evaluation of the prediction performance in the internal and external validation sets. **b** Cross-validation statistics of LASSO-regression model for selection of metabolic biomarkers. Numbers above indicate the average number of selected biomarkers under a given λ. The red dotted line indicates the mean values of AUC under the corresponding λ. The gray error band indicates the minimum and maximum values of AUC. The dashed line points to the optimal model with 27 selected biomarkers. AUC, area under the receiver operating characteristic (ROC) curve. **c** Fold change of 27 selected metabolites in LA group compared with BN group in the discovery set. Red columns, upregulated. Blue columns, downregulated. **d–f** The receiver operating characteristic (ROC) curves showing the efficacy of the discriminant model based on the combination of 27 metabolites in the discovery, internal and external validation sets. Source data are provided as a Source Data file.

As the workflow shown in Fig. 2a, based on the 33 identified differential metabolites in LA (*n* = 136) vs BN (*n* = 170), an optimal variable combination of biomarkers for nodule classification were obtained by least absolute shrinkage and selection operator (LASSO)- binary logistic regression model. A ten-fold cross validation was used to test the robustness of the model. Variable selection and parameter regularization were adjusted by the penalization in likelihood maximization through parameter λ^24^. The global metabolomic analysis was further performed independently in an internal validation (*n* = 104) and an external validation (*n* = 111) cohort to verify the classification performance of the discriminant model. As a result, a panel of 27 metabolites was identified in the discovery set as the best discriminant model with maximum of the mean AUC (Fig. 2b), including 9 upregulated and 18 downregulated in LA compared with BN (Fig. 2c).

A prediction model was created based on these 27 metabolites weighted by their regression coefficient (Supplementary Table 3). The ROC analysis according to these 27 metabolites obtained an area under the curve (AUC) value of 0.933, as well as 0.868 sensitivity and 0.859 specificity in the discovery set (Fig. 2d). Meanwhile, among the 38 annotated differential metabolites between LA and HC, a panel of 16 metabolites achieved an AUC of 0.902 in differentiating between LA and HC with a sensitivity of 0.801 and a specificity of 0.856 (Supplementary Fig. 6a–c). The AUC values based on different fold-change thresholds of differential metabolites were also compared. We found that the classification model yielded the optimal performance to distinguish between LA and BN (HC) when fold change level was set at 1.2 compared with 1.5 or 2.0 (Supplementary Fig. 7a,b). The classification model based on the 27-metabolite panel was further verified in an internal and external cohort. The AUC was 0.915 (0.867 sensitivity, 0.811 specificity) in the internal validation and 0.945 (0.810 sensitivity, 0.979 specificity) in the external validation, respectively (Fig. 2e,f). To evaluate the cross-laboratory performance, 40 samples from the external cohort were analyzed in an outside lab as described in Methods. The classification accuracy reached an AUC of 0.925 (Supplementary Fig. 8). As lung squamous cell carcinoma (LUSC) is the second most common subtype of non-small cell lung cancer (NSCLC) after lung adenocarcinoma (LUAD), we also tested the potential applicability of the verified metabolic signature in a cohort composed of 74 cases of BN and 16 cases of LUSC. The AUC was 0.776 for discrimination of LUSC vs BN (Supplementary Fig. 9), indicating less powerful capacity compared with discrimination of LUAD vs BN.

### Diagnostic capacity of the serum metabolic classifier for nodules in the same size range

**Fig. 3:**
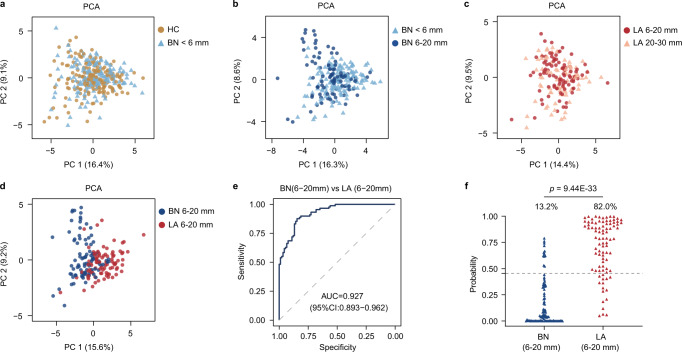
Prediction efficacy of the serum metabolic classifier for nodules in the same size range. **a–d** Comparison of PCA profiles between indicated groups based on the metabolic classifier of 27 metabolites. **a** HC vs BN < 6 mm. **b** BN < 6 mm vs BN 6–20 mm. **c** LA 6–20 mm vs LA 20–30 mm. **d** BN 6–20 mm vs LA 6–20 mm. HC, *n* = 174; BN < 6 mm, *n* = 153; BN 6–20 mm, *n* = 91; LA 6–20 mm, *n* = 89; LA 20–30 mm, *n* = 77. **e** The receiver operating characteristic (ROC) curve showing the efficacy of the discriminant model for nodules 6–20 mm. **f** The probability value calculated from logistic regression model for nodules 6–20 mm. The gray dashed line represents the optimal cut-off value (0.455). Numbers above indicating percentage of cases predicted as LA. Two-sided Student’s *t* test was used. PCA, Principal component analysis. AUC area under the curve. Source data are provided as a Source Data file.

It has been shown that nodule size on CT image positively correlates with probability of malignancy and remains a major determinant for nodule management^25–27^. Data analysis from the large cohort of NELSON screening trial have suggested that the malignancy risk in subjects with nodules <5 mm is even similar to subjects without nodules^28^. Accordingly, the minimum size threshold for the need of routine CT follow-up is 5 mm recommended by the British Thoracic Society (BTS) and 6 mm recommended by the Fleischner Society^29^. However, nodules greater than 6 mm without clear benign features, termed indeterminate pulmonary nodules (IPN), remain a major challenge for evaluation and management in clinical practice^30,31^. With the combined samples from both discovery and internal validation cohort, we further investigated whether there is impact of nodule size on the metabolomic profile. Focusing on the panel of 27 validated biomarkers, we first compared the PCA of metabolomic profiles of HC and BN below 6 mm. We found overlap of most data points from HC and BN, demonstrating similarities in the serum metabolite contents from two groups (Fig. 3a). The signature profiles remained conserved across different size ranges in both BN and LA (Fig. 3b,c), whereas a separation was observed between malignant and benign nodules ranging 6–20 mm (Fig. 3d). The cohort achieved an AUC of 0.927 with 0.868 specificity and 0.820 sensitivity in predicting malignancy of nodules ranging from 6 to 20 mm (Fig. 3e,f). Our results suggest that the classifier may capture the metabolic alterations induced by the malignant transformation at the early-stage, regardless of the nodule size.

**Fig. 4:**
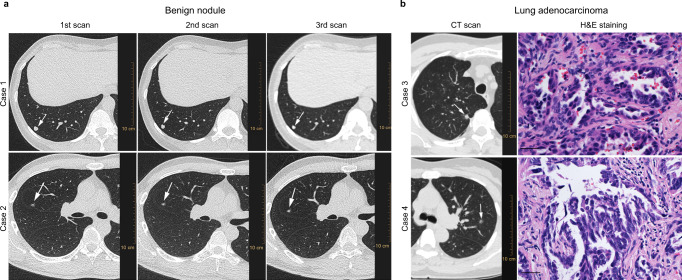
Clinical evaluation of four representative samples diagnosed by the serum metabolic classifier. **a** CT images on axial lung window views of two cases from benign nodules. Case 1 showing a 7 mm stable solid nodule with calcification in the right lower lobe of follow-up CT over 4 years. Case 2 showing a 7 mm stable part-solid nodule in the right upper lobe of follow-up CT over 5 years. **b** CT images on axial lung window views before lung resection surgery and the matched pathological examination of two cases of stage I adenocarcinoma. Case 3 showing an 8 mm nodule with pleural retraction int the right upper lobe. Case 4 showing a 9 mm part-solid ground glass nodule in the left upper lobe. Haematoxyline and Eosin (H&E) staining of lung tissue from resection surgery (scale bar = 50 μm) showing acinar growth pattern of lung adenocarcinoma. Arrows indicate nodules detected on CT images. The H&E images are representative views of multiple (>3) microscopic fields examined by the pathologist.

4 samples (aged 44-61) with pulmonary nodules that were similar in size (7–9 mm), were further selected to illustrate the performance of the proposed model for malignancy prediction (Fig. 4a,b). At the initial screening, case 1 represents a solid nodule with calcification, which is a feature associated with benign nature, while case 2 represents an indeterminate part-solid nodule without clear benign features. Three rounds of follow-up CT demonstrated that these cases remained stable over a 4-year-period of time and therefore were considered benign nodules (Fig. 4a). Compared with the clinical evaluation by serial CT scans, a single analysis of serum metabolites by the current classifier model rapidly and correctly identified these benign nodules based on the probability cut-off value (Table 1). In Fig. 4b, case 3 presents a nodule with a pleural retraction feature which is more commonly associated with malignancy^32^. Case 4 presents an indeterminate part-solid nodule without signs of benign causes. These cases were both predicted to be malignant by the classifier model (Table 1). The evaluation of lung adenocarcinoma was proved by histopathological examination after lung resection surgery (Fig. 4b). For the external validation set, two cases of indeterminate pulmonary nodules above 6 mm were presented with accurate prediction by the metabolic classifier (Supplementary Fig. 10).

**Table 1:** Prediction probability values and outcomes of the four representative pulmonary nodules by the serum metabolic classifier

	Case 1	Case 2	Case 3	Case 4
Gender	F	M	F	M
Maximal diameter (mm)	7	7	8	9
Probability	0.330	0.003	0.492	0.887
Prediction	BN	BN	LA	LA

Taken together, our findings suggest the potential value of the serum metabolite biomarkers in differential diagnosis of pulmonary nodules that may pose challenges for evaluation by CT screening.

### Perturbation of tryptophan metabolism is associated with active glycolysis in lung adenocarcinoma

**Fig. 5:**
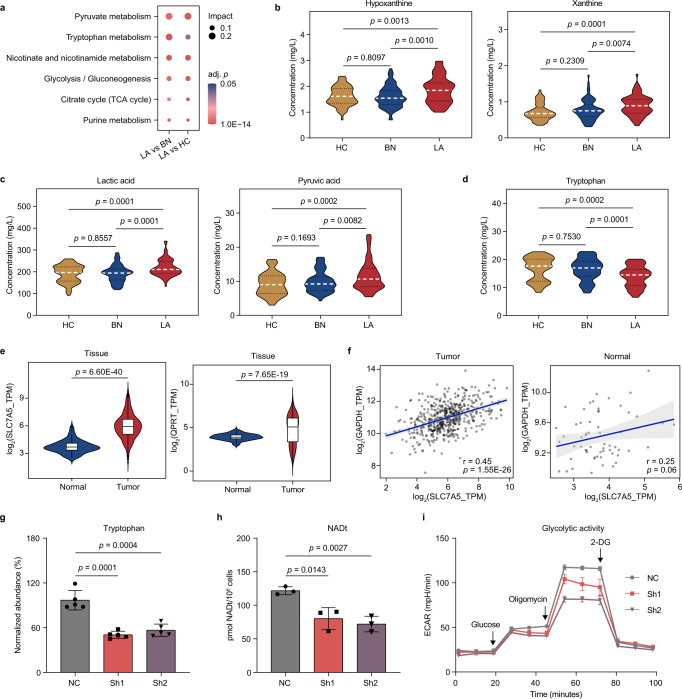
Correlation between tryptophan metabolism and glycolytic activity in lung adenocarcinoma. **a** KEGG pathway enrichment analysis of significantly differential metabolites in LA group compared with BN and HC. Two-sided Globaltest was used and *p* values was adjusted by Holm-Bonferroni method (adj. *p* ≤ 0.001 and impact > 0.01). **b–d** The violin plots showing levels of hypoxanthine, xanthine, lactate, pyruvate, and tryptophan in the serum of HC, BN, and LA determined by LC-MS/MS (*n* = 70 in each group). The white and black dashed lines indicate the medians and quartiles, respectively. **e** The violin plots showing normalized Log_2_TPM (transcript per million) mRNA expression of SLC7A5 and QPRT in lung adenocarcinoma (*n* = 513) vs normal lung tissues (*n* = 59) in the LUAD-TCGA dataset. The white box represents the interquartile range, the horizontal black line in the center indicates the median and the vertical black line extended from the box indicates 95% confidence intervals (CI). **f** The Pearson’s correlation plot of SLC7A5 with GAPDH expression in lung adenocarcinoma (*n* = 513) and normal lung tissues (*n* = 59) from TCGA dataset. The gray area represents 95% CI. r, Pearson’s correlation coefficient. **g** Normalized cellular levels of tryptophan in A549 cells transfected with shRNA-nonspecific control (NC) and shSLC7A5 (Sh1, Sh2) were determined by LC-MS/MS. Statistical analysis is shown with five biologically independent samples in each group. **h** Cellular levels of NADt (NAD total including NAD^+^ and NADH) in A549 cells (NC) and A549 cells with knockdown of SLC7A5 (Sh1, Sh2). Statistical analysis is shown with three biologically independent samples in each group. **i** Glycolytic activity of A549 cells before and after knockdown of SLC7A5 was measured by the extracellular acidification rate (ECAR) (*n* = 4 biologically independent samples in each group). 2-DG, 2-deoxy-D-glucose. Two-sided Student’s *t* test was used in (**b**–**h**). In (**g**–**i**), error bars represent means ± S.D and each experiment was performed three times independently with similar results. Source data are provided as a Source Data file.

Based on the verified panel of differential metabolites, we sought to determine the biological relevance of the major metabolic alterations. KEGG pathway enrichment analysis by MetaboAnalyst revealed 6 common significantly altered pathways between two given groups (LA vs HC and LA vs BN, adj. *p* ≤ 0.001, impact > 0.01). These alterations were characterized by perturbation of pyruvate metabolism, tryptophan metabolism, nicotinate and nicotinamide metabolism, glycolysis, TCA cycle, and purine metabolism (Fig. 5a). We then further pursued targeted metabolomics to verify the major alterations with absolute quantification. The shared metabolites in the commonly altered pathways were determined by triple quadrupole mass spectrometer (QQQ) using authentic metabolite standards. The demographic characteristics of the samples for targeted metabolomic study were included in Supplementary Table 4. Consistent with our findings from global metabolomics, the quantitative analysis confirmed elevation of hypoxanthine and xanthine, pyruvate and lactate in LA compared with BN and HC (Fig. 5b,c, *p* < 0.05). Meanwhile, no significant difference of these metabolites was detected between BN and HC.

Given the significant impact of tryptophan metabolism alteration in the LA group, we also evaluated the serum level of tryptophan by QQQ in HC, BN and LA groups. We found decreased abundance of serum tryptophan in LA compared with either HC or BN (*p* < 0.001, Fig. 5d), which was in line with previous findings of lower circulating tryptophan in lung cancer patients compared with healthy controls^33–35^. Another study using a PET/CT tracer of ^11^C-methyl-L-tryptophan demonstrated substantially prolonged retention of tryptophan signal in the lung cancer tissue compared with the benign lesion or normal tissue^36^. We reasoned that the decrease of tryptophan in serum of LA may reflect the active uptake of tryptophan into the lung cancer cells.

It is also known that the final product of kynurenine pathway in tryptophan catabolism is NAD^+^^37,38^, which is an obligatory substrate for the reaction of glyceraldehyde-3-phosphate to 1,3-diphosphoglycerate in glycolysis^39^. While previous studies have focused on the role of tryptophan catabolism in immune regulation^40–42^, we sought to elucidate the interaction between the dysregulated tryptophan and glycolytic pathways observed in the current study. Solute Carrier Family 7 Member 5 (SLC7A5) has been known to be a tryptophan transporter^43–45^. Quinolinate phosphoribosyltransferase (QPRT) is the enzyme that converted quinolinic acid into NAMN in the downstream of kynurenine pathway^46^. Examination of TCGA dataset of LUAD revealed that SLC7A5 and QPRT are both significantly upregulated in the tumor tissues compared with the normal tissues (Fig. 5e). Such elevation was observed in both stage I&II and stage III&IV lung adenocarcinoma (Supplementary Fig. 11), indicating early aberration in tryptophan metabolism associated with tumorigenesis.

Furthermore, the LUAD-TCGA dataset showed a positive correlation between SLC7A5 and GAPDH mRNA expression in the cancer patient samples (*r* = 0.45, *p* = 1.55E−26, Fig. 5f). In contrast, no significant correlation was found between such gene signatures in the normal lung tissues (*r* = 0.25, *p* = 0.06, Fig. 5f). Knockdown of SLC7A5 (Supplementary Fig. 12) in A549 cells significantly reduced cellular levels of tryptophan and NAD(H) (Fig. 5g,h), leading to attenuation of glycolytic activity which was measured by extracellular acidification rate (ECAR) (Fig. 5i). Therefore, based on the serum metabolic alterations and in vitro assays, we proposed that tryptophan metabolism may play an important role in promoting glycolysis in lung cancer via production of NAD^+^ by the kynurenine pathway.

**Fig. 6:**
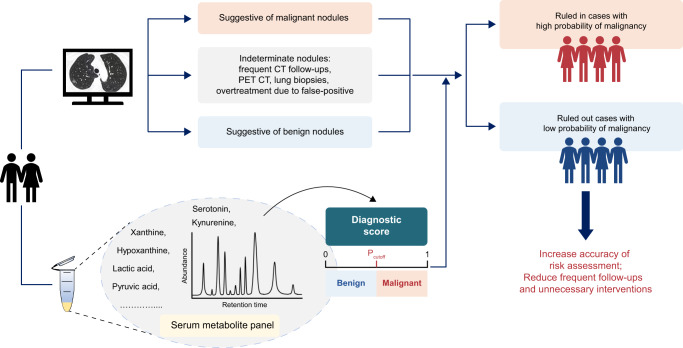
Proposed strategy of pulmonary nodule classification combined with CT screening. Pulmonary nodules are evaluated by low-dose computed tomography (LDCT) with imaging features suggestive of benign or malignant causes. The outcome of indeterminate nodules may lead to frequent follow-ups, unnecessary interventions and overtreatment. Incorporating a serum metabolic classifier with diagnostic value potentially enhances the risk assessment and improves the subsequent management of pulmonary nodules. PET positron emission tomography.

It has been shown that a large number of indeterminate pulmonary nodules detected by LDCT may cause additional evaluation such as PET CT, lung biopsies and overtreatment due to false positive diagnosis of malignancy^31^. As illustrated in Fig. 6, our study revealed a panel of serum metabolites with potential diagnostic value, which may improve risk stratification and follow-up management of pulmonary nodules detected by CT screening.

## Discussion

Evidence from the American NLST trial as well as the European NELSON trial has demonstrated that screening with low-dose computed tomography (LDCT) in high-risk individuals reduces mortality of lung cancer^1,3^. However, risk assessment and subsequent clinical management of the large number of incidental pulmonary nodules detected by LDCT remain most challenging. A critical goal is to optimize the proper classification of the current LDCT-based protocol by incorporating reliable biomarkers.

Certain molecular biomarkers such as blood-based metabolites have been identified by comparing lung cancer with healthy controls^15,17^. In the current study, we focused on the utility of serum metabolomic profiling for discriminating between benign and malignant pulmonary nodules incidentally detected by LDCT. We compared the global serum metabolomes of samples from healthy controls (HC), benign pulmonary nodules (BN) and stage I lung adenocarcinoma (LA) by UPLC-HRMS analysis. We found that HC and BN share similarities in metabolite features while LA displayed significant alterations compared with HC and BN. We identified two panels of serum metabolites to distinguish LA from HC and BN.

The current LDCT-based protocol of discrimination between benign and malignant nodules is mainly based on the nodule size, density, morphology and growth rate over time^30^. Previous studies indicate that nodule size is strongly associated with probability of lung cancer. The risk of malignancy is <1% in nodules <6 mm even in patients at high-risk. The risk of malignancy for nodules between 6 mm and 20 mm can range from 8 to 64%^30^. Therefore, a cut-off diameter of 6 mm on CT imaging is recommended by the Fleischner society for routine follow-up^29^. However, risk assessment and management of indeterminate pulmonary nodules (IPN) over 6 mm have not been well-performed^31^. Current IPN management is usually based on watchful waiting for frequent CT follow-ups.

Based on the validated metabolite panel, we first demonstrated an overlap of metabolomic profiles between healthy controls and benign nodules <6 mm. The biological similarities coincide with previous CT findings that the risk of malignancy for nodules <6 mm is as low as those of subjects without nodules^30^. Notably, our results further showed that benign nodules <6 mm and ≥6 mm shared major similarities in metabolomic profiles, indicating a uniform functional readout of benign etiology regardless of nodule size. As such, the current diagnostic panel of serum metabolites may provide a single analysis as a rule-out test at initial discovery of nodule on CT and potentially reduce serial surveillance. Meanwhile, the same metabolic biomarker panel separates malignant from benign nodules ≥6 mm and provides accurate prediction for IPNs with similar sizes and undetermined indication of morphology on CT images. This serum metabolic classifier achieves a well-performed AUC of 0.927 in predicting malignancy of nodules ≥6 mm. Collectively, our findings suggest that the distinct serum metabolomic signature may specifically reflect the tumor-derived metabolic alterations at the early stage and present the potential value as a risk predictor independent of nodule size.

It should be noted that lung adenocarcinoma (LUAD) and squamous cell carcinoma (LUSC) are predominant types of non-small cell lung cancer (NSCLC). Considering that LUSC is highly associated with tobacco use^47^, and LUAD is the most frequent histology of incidental pulmonary nodules detected by CT screening^48^, our classifier model is specifically established with samples of stage I adenocarcinoma. Wang and colleagues have also focused on LUAD and identified nine lipid features by lipidomics to differentiate early-stage lung cancer from healthy controls^17^. We have tested the current classifier model with 16 cases of stage I LUSC and 74 cases of benign nodule and observed less accuracy in predicting LUSC (AUC 0.776), suggesting that LUAD and LUSC may have their own metabolomic characteristics. Indeed, it has been shown that LUAD and LUSC are different in etiology, biologic origins and genetic aberrations^49^. Therefore, other types of histology shall be included in a training model for detection of lung cancer in the general population of a screening program.

Here, we revealed six commonly altered pathways in lung adenocarcinoma compared with healthy controls and benign nodules. Xanthine and hypoxanthine were the shared metabolites in the purine metabolism pathway. Consistent with our results, intermediates associated with purine metabolism have been found to be markedly increased in the serum or tissue of lung adenocarcinoma patients compared with healthy controls or patient at pre-invasive stage^15,50^. The increased abundance of serum xanthine and hypoxanthine may reflect the anabolism required for building blocks of rapid proliferating cancer cells. Deregulation of glucose metabolism is a renown hallmark of cancer metabolism^51^. Here we observed significant increase of pyruvate and lactate in the LA group compared with HC and BN, which is in line with previous findings of perturbation in glycolytic pathway in serum metabolome profiling of non-small cell lung cancer (NSCLC) patients compared with healthy controls^52,53^.

Importantly, we observed inverse correlation between pyruvate and tryptophan metabolism in serum of lung adenocarcinoma. Serum tryptophan level was decreased in the LA group compared with HC or BN. Interestingly, previous work using a large-scale study from prospective cohorts suggests that low level of circulating tryptophan was associated with increased risk of lung cancer^54^. Tryptophan is an essential amino acid exclusively obtained from dietary uptake. We reasoned that depletion of serum tryptophan in lung adenocarcinoma likely reflects rapid consumption of this metabolite. It is known that the end product of tryptophan catabolism through kynurenine pathway is a source for de novo synthesis of NAD^+^. As NAD^+^ is mostly produced by salvage pathway, the relevance of NAD^+^ from tryptophan metabolism in health and disease remains to be established^46^. Our analysis of TCGA database showed that expression of the tryptophan transporter, solute carrier 7A5 (SLC7A5) is significantly upregulated in lung adenocarcinoma compared with normal controls and positively correlates with expression of glycolytic enzyme GAPDH. Previous studies have focused on the role of tryptophan catabolism in suppressing antitumor immune response^40–42^. Here, we demonstrated that inhibition of tryptophan uptake by knockdown of SLC7A5 in the lung cancer cell caused subsequent decrease of cellular level of NAD, along with attenuation of glycolytic activity. Therefore, our study provides a biological basis for the serum metabolic alteration associated with malignant transformation in lung adenocarcinoma.

EGFR mutations represent the most common actionable driver mutations in patients with NSCLC. In our study, we found that EGFR-mutant patients (*n* = 41) shared similar global metabolomic profiles with EGFR-wild type patients (*n* = 31), although we identified a number of acylcarnitines with decreased abundance in the serum of EGFR-mutant patients. The established function of acylcarnitines is to transport acyl groups from the cytosol to mitochondrial matrix, leading to fatty acid oxidation for energy production^55^. In line with our findings, a recent study has also observed comparable metabolomic profiles between EGFR-mutant and EGFR-wild type tumors by analyzing global metabolomes of 102 samples of lung adenocarcinoma tissues^50^. Interestingly, an acylcarnitine was also discovered with decreased abundance in the EGFR-mutant group. Therefore, whether the change of acylcarnitine levels reflects the EGFR-driven metabolic alterations and the underlying molecular links may be worthy of further investigation.

In summary, our study has established a serum metabolic classifier for differential diagnosis of pulmonary nodules, and proposed a workflow to potentially optimize the risk assessment and facilitate clinical management based on computed tomography screening.

## Methods

### Collection of clinical samples

The study was approved by the Ethics Committee of Sun Yat-sen University Cancer Center, The First Affiliated Hospital of Sun Yat-sen University, and The Affiliated Cancer Hospital of Zhengzhou University. In the discovery and internal validation cohorts, serum of 174 cases of healthy controls and 244 cases of benign nodules were collected from individuals undergoing annual physical examination at Department of Cancer Prevention and Medical Examination, Sun Yat-sen University Cancer Center, and 166 cases of stage I lung adenocarcinoma were collected at Sun Yat-sen University Cancer Center. In the external validation cohorts, 48 cases of benign nodules and 39 cases of stage I lung adenocarcinoma were collected from The First Affiliated Hospital of Sun Yat-sen University, and 24 cases of state I lung adenocarcinoma were collected from The Affiliated Cancer Hospital of Zhengzhou University. 16 cases of stage I lung squamous cell carcinoma were also collected from Sun Yat-sen University Cancer Center to test the diagnostic capacity of the established metabolic classifier (patient characteristics in Supplementary Table 5). Samples for the discovery and internal validation cohorts were collected between January 2018 and May 2020. Samples for the external validation cohort were collected between August 2021 and October 2022. To minimize gender bias, an approximately equal number of male and female cases were assigned to each group of the discovery and internal validation cohorts. Gender of participants was determined based on self-report. Informed consents were obtained from all participants and no compensation was provided. Subjects of benign nodules were those with stable evaluations by follow-up CT scans over 2–5 years at the time of analysis, except that one case in the external validation set was collected prior to surgery and diagnosis of chronic bronchitis was made by histopathological examination. Lung adenocarcinoma cases were collected prior to lung resection surgery and confirmed by pathological diagnosis. Fasting blood samples were collected with the serum separator tube without any anticoagulants. The blood samples were clotted at room temperature within 1 h and then centrifuged at 2851 × *g* for 10 min at 4 °C to collect serum supernatant. The serum aliquots were frozen at −80 °C until metabolite extraction. A serum pool was collected from 100 healthy donors at Department of Cancer Prevention and Medical Examination, Sun Yat-sen University Cancer Center, with an equal number of men and women aged from 40 to 55. Equal volumes of each donor samples were mixed and the resulting pool was dispensed in aliquots and stored at −80 °C. The serum mixture was used as a reference material for quality control and data normalization.

### Serum metabolite extraction

Reference serum and study samples were thawed and metabolites were extracted using the combined extraction method (MTBE/methanol/water)^56^. Briefly, 50 μL serum was mixed with 225 μL ice-cold methanol and 750 μL ice-cold methyl-tertbutyl ether (MTBE). The mixture was vortexed and incubated on ice for 1 h. The samples were then mixed with 188 μL MS-grade water containing internal standards (^13^C-lactate, ^13^C_3_-pyruvate, ^13^C-methionine and ^13^C_6_-isoleucine were purchased from Cambridge Isotope Laboratories) and vortexed. The mixture was then centrifuged at 15,000 × *g* at 4 °C for 10 min and the bottom phase was transferred to two tubes (125 μL/each tube) for positive and negative mode analysis of LC-MS. Finally, the samples were evaporated to dryness under a speed vacuum concentrator.

### Untargeted liquid chromatography-mass spectrometry analysis

Dried metabolites were reconstituted in 120 μL 80% acetonitrile, vortexed for 5 min, and centrifuged at 15,000 × *g* at 4 °C for 10 min. The supernatant was transferred to a glass amber vial with micro-insert for the metabolomic study. Untargeted metabolomic analysis was performed on an ultra-performance liquid chromatography-high resolution mass spectrometry (UPLC–HRMS) platform. The metabolites were separated by Dionex Ultimate 3000 UPLC system with ACQUITY BEH Amide column (2.1 × 100 mm, 1.7 μm, Waters). In positive mode, the mobile phases were 95% (A) and 50% acetonitrile (B), with 10 mmol/L ammonium acetate and 0.1% formic acid in both phases. In negative mode, mobile phase A and B were 95 and 50% acetonitrile, containing 10 mmol/L ammonium acetate with pH = 9 in both phases. The gradient program was as follows: 0–0.5 min, 2% B; 0.5–12 min, 2–50% B; 12–14 min, 50–98% B; 14–16 min, 98% B; 16–16.1 min, 98–2% B; 16.1–20 min, 2% B. The column was maintained at 40 °C and the samples were kept in the autosampler at 10 °C. The flow rate was 0.3 mL/min, and the injection volume was 3 μL. A Q-Exactive orbitrap mass spectrometer (Thermo Fisher Scientific) with electrospray ionization (ESI) source was operated in full scan mode combined with ddMS2 monitoring mode for mass data acquisition. The MS parameters were set as follows: spray voltage +3.8 kV/− 3.2 kV; capillary temperature 320 °C; sheath gas 40 arb; auxiliary gas 10 arb; probe heater temperature 350 °C; scan range 70–1050 m/z; resolution 70,000. The data were acquired using Xcalibur 4.1 (Thermo Fisher Scientific).

For data quality assessment, pooled quality control (QC) samples were created by taking 10 μL aliquot supernatant from each sample. Six injections of QC samples were analyzed at the beginning of the analytical sequence for assessing the stability of UPLC-MS system. Then QC samples were injected periodically throughout the whole batch. All serum samples were completed for LC-MS analysis in 11 batches in the current study. Aliquots of serum pool mixture from 100 healthy donors were used as reference material in corresponding batch to monitor the extraction process and correct the inter-batch effects. The untargeted metabolomic analysis of discovery cohort, internal and external validation cohort were performed at Sun Yat-sen University Metabolomics Center. 40 samples from the external cohort were also analyzed in an outside lab at Analysis and Test Center, Guangdong University of Technology to test the performance of the classifier model.

### Targeted metabolomics

After extraction and reconstitution, the absolute quantitation of serum metabolites was measured by ultra-performance liquid chromatography- tandem mass spectrometry (Agilent 6495 Triple Quadrupole) with electrospray ionization (ESI) source operated in multiple reaction monitoring (MRM) mode. The ACQUITY BEH Amide column (2.1 × 100 mm, 1.7 μm, Waters) was used to separate metabolites. The mobile phases were consisted of 90% (A) and 5% acetonitrile (B) both with 10 mmol/L ammonium acetate and 0.1% ammonia solution. The gradient program was as follows: 0–1.5 min, 0% B; 1.5–6.5 min, 0–15% B; 6.5–8 min, 15% B; 8–8.5 min, 15%–0% B; 8.5–11.5 min, 0% B. The column was maintained at 40 °C and the samples were kept in the autosampler at 10 °C. The flow rate was 0.3 mL/min, and the injection volume was 1 μL. The MS parameters were set as follows: capillary voltage ±3.5 kV; nebulizer pressure 35 psi; sheath gas flow rate 12 L/min, sheath gas temperature 350 °C, dry gas temperature 250 °C, and dry gas flow rate 14 L/min. The MRM transitions for tryptophan, pyruvate, lactate, hypoxanthine, and xanthine were 205.0–187.9, 87.0–43.4, 89.0–43.3, 135.0–92.3 and 151.0–107.9, respectively. The data were acquired by Mass Hunter B.07.00 (Agilent Technologies). For serum samples, tryptophan, pyruvate, lactate, hypoxanthine, and xanthine were quantified by the calibration curves of standard mixture solution. For cell samples, the abundance of tryptophan was normalized by the internal standard and the protein mass of cells.

### Metabolomic data processing

Peak extraction with *m/z* and retention time (RT) was performed by Compound Discovery 3.1 and TraceFinder 4.0 (Thermo Fisher Scientific). To remove potential inter-batch variations, each feature peak of study samples was divided by that of reference material from the same batch to obtain the relative abundance. Relative standard deviation of internal standards before and after normalization was shown in Supplementary Table 6. Differential features between two groups were selected by the *p* value adjusted by false discovery rate (FDR < 0.05, Wilcoxon rank tests) and fold change (>1.2 or <0.83). The raw MS data of extracted features and the corrected MS data by reference serum were shown in Supplementary data 1 and Supplementary data 2, respectively. Peak annotation was conducted according to the four defined levels of identification, including identified metabolites, putatively annotated compounds, putatively characterized compound classes, and unknown compounds^22^. Based on database searching (mzCloud, HMDB, Chemspider) in Compound Discovery 3.1, biological compounds with MS/MS matched to authenticated standards, or putatively annotated by full match in mzCloud (score > 85) or Chemspider were finally selected as differential metabolites between two groups. The peak annotation of each feature is included in Supplementary Data 3. MetaboAnalyst 5.0 was used for univariate analysis of the metabolite abundance normalized by sum. KEGG pathway enrichment analysis was also assessed by MetaboAnalyst 5.0 based on significantly differential metabolites. The principal component analysis (PCA) and partial least squares discrimination analysis (PLS-DA) were analyzed by ropls package (v.1.26.4) with sum normalization and auto scaling. The optimal metabolite biomarker model for predicting nodule malignancy was built by binary logistic regression with the least absolute shrinkage and selection operator (LASSO, R package v.4.1-3). The performance of the discriminant model in both discovery and validation set was characterized by estimating AUC according the ROC analysis by pROC package (v.1.18.0.). The optimal probability cut-off value was obtained according to the maximum Youden Index (sensitivity +specificity-1) of the model. Samples with values less or greater than cut-off value will be predicted as benign nodules and lung adenocarcinoma, respectively.

### Cell culture and knockdown of SLC7A5

A549 cells (#CCL-185, American Type Culture Collection) were grown in F-12K medium with 10% FBS. Short hairpin RNA (shRNA) sequences targeting SLC7A5 and non-targeting control (NC) were inserted into the pLKO.1-puro lentiviral vector. Anti-sense sequences for shSLC7A5 were as follows: Sh1 (5′- GGAGAAACCTGATGAACAGTT −3′), Sh2 (5′-GCCGTGGACTTCGGGAACTAT-3′). Antibody for SLC7A5 (#5347) and tubulin (#2148) were purchased from Cell Signaling Technology. The antibody dilution of SLC7A5 and tublin is 1:1000 for westernblot analysis.

### Glycolytic activity

Seahorse XF Glycolysis Stress Test was used to measure the extracellular acidification rate (ECAR). In the assay, glucose, oligomycin A and 2-DG were sequentially injected to test the cellular glycolytic capacity measured by ECAR.

### Measurement of cellular tryptophan and NAD(H)

A549 cells transfected with non-targeting control (NC) and shSLC7A5 (Sh1, Sh2) were plated into 10-cm dishes overnight. Cellular metabolites were extracted with 1 ml iced-cold 80% methanol aqueous solution. Cells in methanol solution were scratched, collected in a new tube, and centrifuged at 15,000 × *g* for 15 min at 4 °C. 800 μL supernatant was collected and dried using a speed vacuum concentrator. The dried metabolite pellets were then analyzed for tryptophan level by LC-MS/MS as described above. Cellular NAD(H) levels of A549 cells (NC and shSLC7A5) were measured by NAD^+^/NADH Quantification Colorimetric Kit (#K337, BioVision) as instructed by the manufacturer. Protein levels of each sample were measured to normalized the metabolite amount.

### Statistics and reproducibility

No statistical method was used to predetermine sample size. Previous metabolomic studies^15,18^ for biomarker discovery were considered as a reference for size determination and our samples were adequate compared with these reports. No samples were excluded in the study cohorts. Serum samples were randomly allocated to the discovery (306, 74.6%) and the internal validation cohorts (104, 25.4%) for untargeted metabolomics study. 70 cases in each group were also randomly selected from the discovery set for targeted metabolomic study. The investigators were blinded to group allocation when performed LC-MS data acquisition and data analysis. Statistical analysis of metabolomics data and cellular experiments were described in the corresponding Results, figure legends and Method sections. The quantification of cellular tryptophan, NADt and glycolytic activity were performed three times independently with similar results.

### Reporting summary

Further information on research design is available in the Nature Portfolio Reporting Summary linked to this article.

## Supplementary information

Supplementary Information

Reporting Summary

Description of Additional Supplementary Files

Supplementary Data 1

Supplementary Data 2

Supplementary Data 3

## Data Availability

The raw MS data of extracted features and the normalized MS data by reference serum are shown in Supplementary data 1 and Supplementary data 2, respectively. Peak annotation of differential features is provided in Supplementary data 3. The LUAD dataset from TCGA can be downloaded from https://portal.gdc.cancer.gov/. Raw data of plotting figures are provided in Source Data. Source data are provided with this paper.

## Source data

Source Data
