# Supplementary material for: Metabolomic differentiation of benign vs malignant pulmonary nodules with high specificity via high-resolution mass spectrometry analysis of patient sera
Source: Nat Commun. 2023 Apr 24;14:2339. doi: 10.1038/s41467-023-37875-1 (PMC10126054; doi:10.1038/s41467-023-37875-1)
Supplement: Supplementary file 1 — Supplementary Information [file 41467_2023_37875_MOESM1_ESM.pdf]

Supplementary Information

Metabolomic differentiation of benign vs malignant pulmonary nodules with high specificity via high-resolution mass spectrometry analysis of patient sera

Yao Yao[1,#], Xueping Wang[2,#], Jian Guan[3,#], Chuanbo Xie[4], Hui Zhang[4,5], Jing Yang[4], Yao Luo[4], Lili Chen[6], Mingyue Zhao[4], Bitao Huo[4,5], Tiantian Yu[5], Wenhua Lu[4], Qiao Liu[4], Hongli Du[7], Yuying Liu[4], Peng Huang[4,5], Tiangang Luan[1,8*], Wanli Liu[2*], Yumin Hu[4,5*]

[1]Sate Key Laboratory of Biocontrol, School of Life Sciences, Sun Yat-sen University, Guangzhou, Guangdong, 510275, China

[2]Department of Clinical Laboratory, State Key Laboratory of Oncology in South China, Sun Yat-sen University Cancer Center, Guangzhou, Guangdong, 510060, China

[3]Department of Radiology, The First Affiliated Hospital of Sun Yat-sen University, Guangzhou, Guangdong, 510080, China

[4]State Key Laboratory of Oncology in South China, Collaborative Innovation Center for Cancer Medicine, Sun Yat-sen University Cancer Center, Guangzhou, Guangdong, 510060, China

[5]Metabolomics Research Center, Zhongshan School of Medicine, Sun Yat-sen University, Guangzhou, Guangdong, 510080, China

[6]Department of Pathology, The First Affiliated Hospital of Sun Yat-sen University, Guangzhou, Guangdong, 510080, China

[7]School of Biology and Biological Engineering, South China University of Technology, Guangzhou, Guangdong, 510006, China

[8]Institute of Environmental and Ecological Engineering, Guangdong University of Technology, Guangzhou, Guangdong, 510006, China

[#]These authors contributed equally.

***Correspondence:** huym@sysucc.org.cn (Y.H.); liuwl@sysucc.org.cn (W.L.); cesltg@mail.sysu.edu.cn (T.L.)

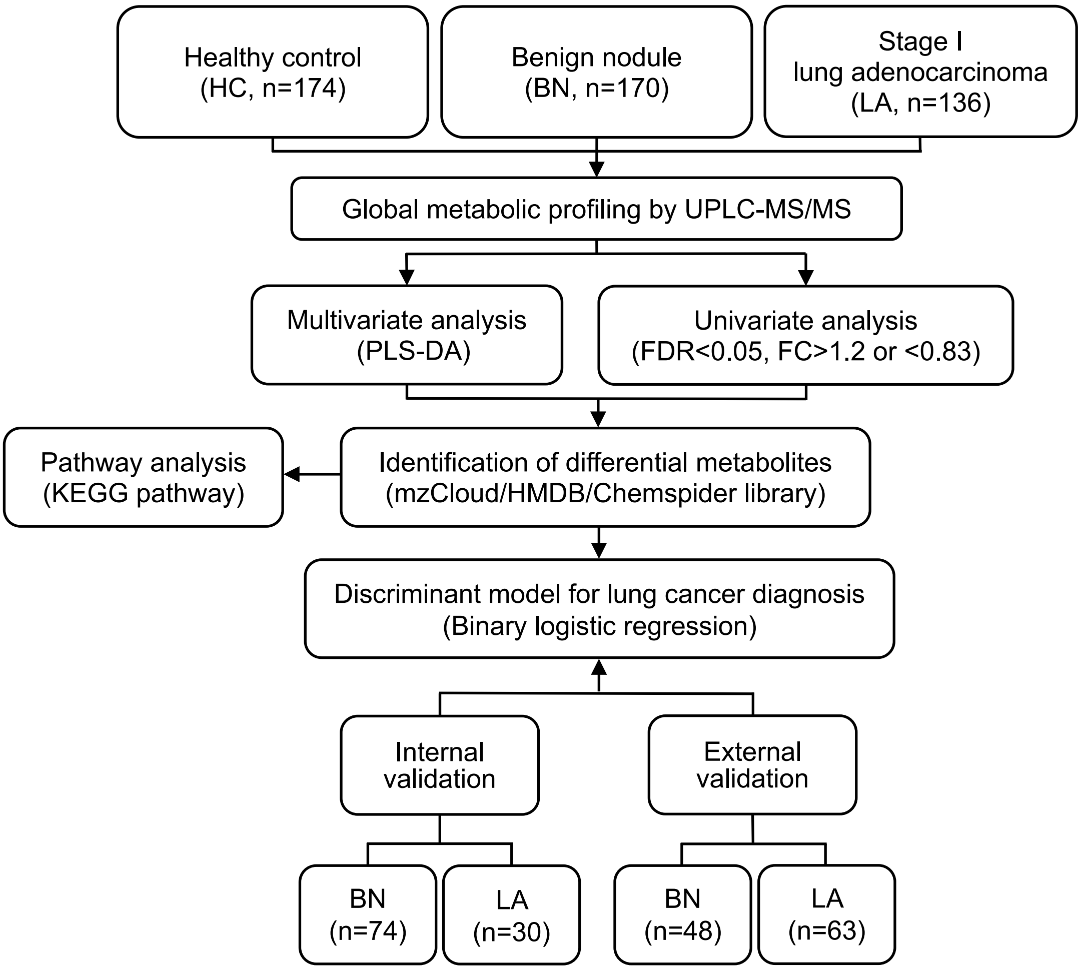

Supplementary Figure 1. Workflow of the study design. A total of 695 serum samples were collected in this study for global metabolomics analysis. 480 samples including 174 healthy controls, 170 benign pulmonary nodules, and 136 stage I lung adenocarcinoma were used in the discovery set for metabolite biomarker selection. A total of 104 and 111 samples were used as internal and external validation, respectively. HC, healthy control BN, benign nodule. LA, lung adenocarcinoma. UPLC-HRMS, ultra-performance liquid chromatography- high resolution mass spectrometry. PLS-DA, Partial least squares discriminant analysis. FC, fold change. FDR, false discovery rate.

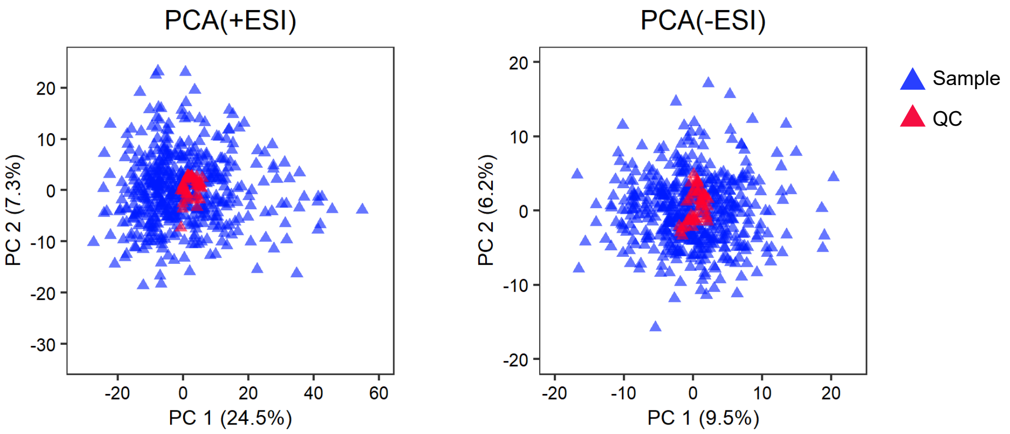

Supplementary Figure 2. Principal component analysis (PCA) of quality control (QC) and experimental samples. Left and right, positive (+ESI) and negative (-ESI) electrospray ionization mode. Experimental samples, n=480; QC, n= 49.

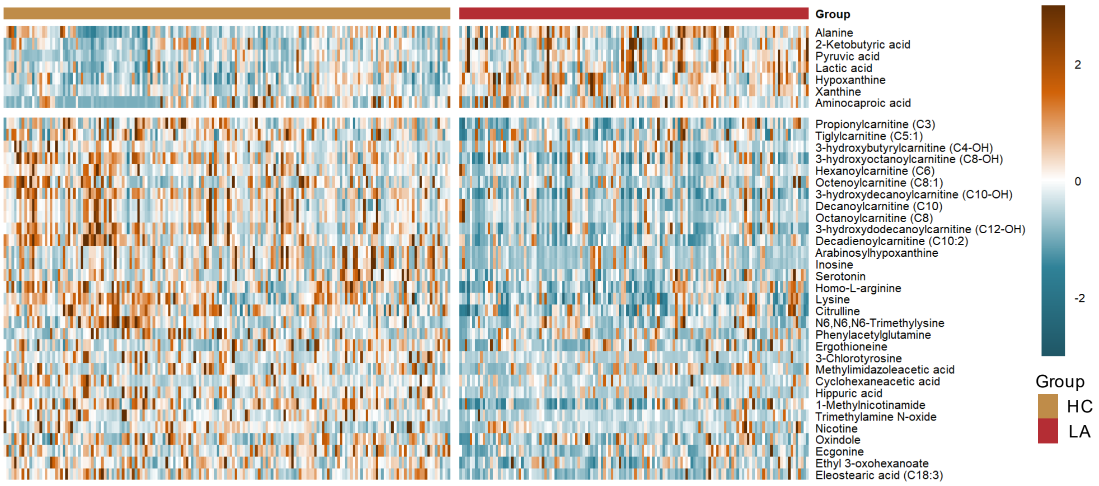

Supplementary Figure 3. A hierarchical clustering heat map showing significantly differential abundance of annotated metabolites between two given groups. 38 differential metabolites were identified between lung adenocarcinoma (LA, n=136) and healthy controls (HC, n=174). Each sample is colored in blue to brown to indicate relative intensity of metabolite abundance.

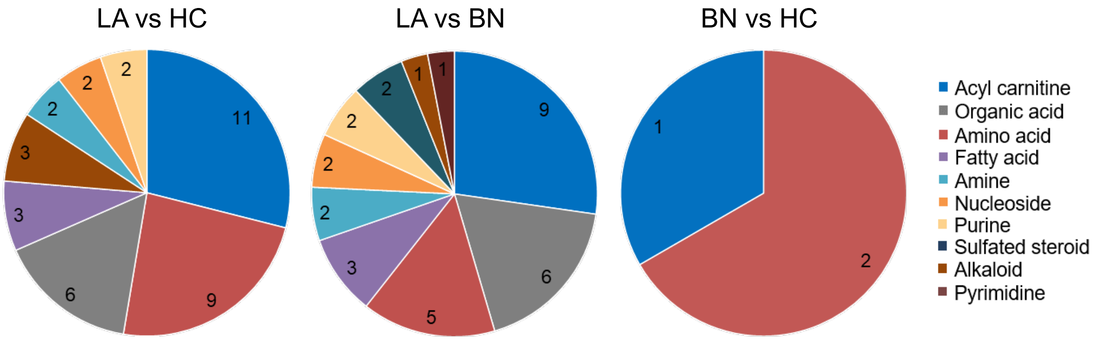

Supplementary Figure 4. Classes and counts of differentially abundant metabolites between two given groups. HC, healthy control; BN, benign nodule; LA, stage I lung adenocarcinoma.

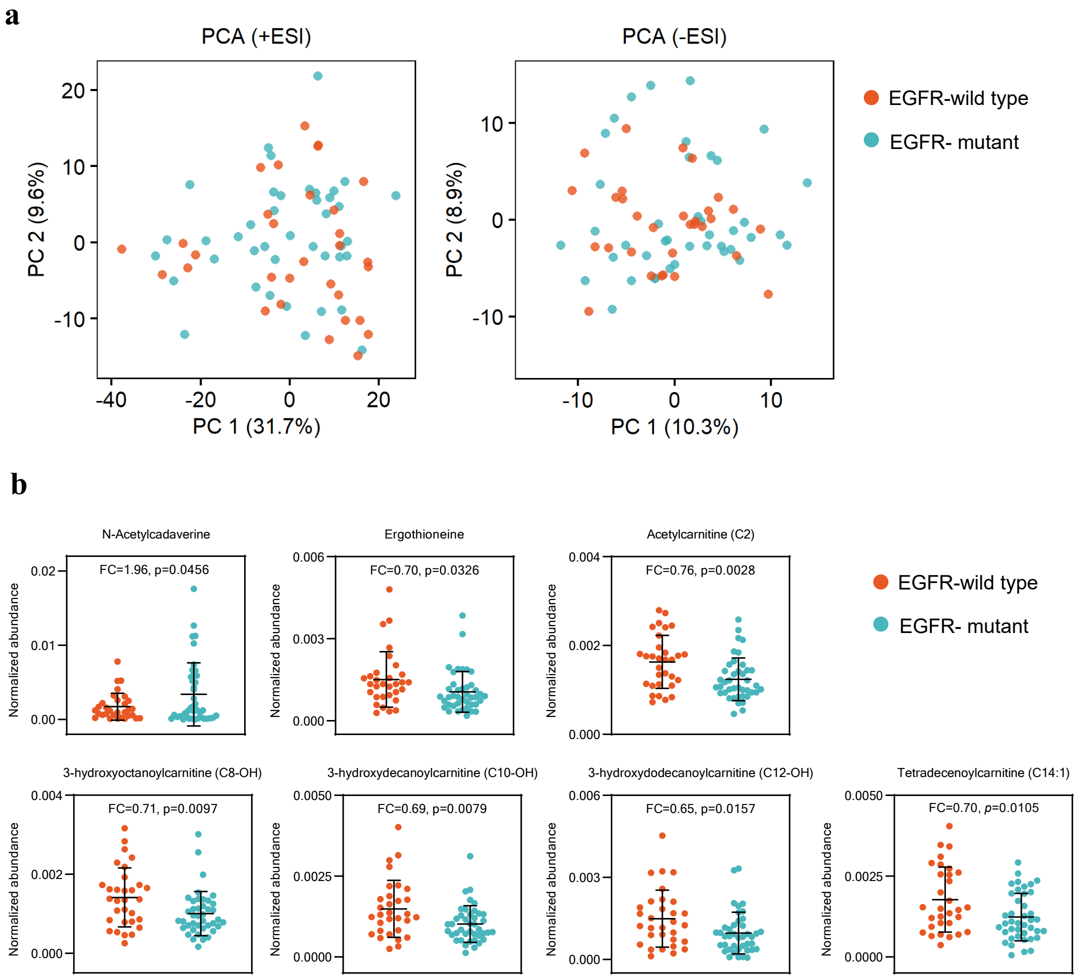

Supplementary Figure 5. Alterations in serum metabolites of EGFR-mutant patients compared with EGFR-wild type patients. a, Principal component analysis (PCA) of serum metabolomes in stage I adenocarcinoma patients (EGFR-mutant, n=41; EGFR-wild type, n=31). **b**, Differential metabolites in EGFR-mutant vs EGFR-wild type (two-sided Student's *t*-test, p<0.05) patients. FC, fold change. Bars, means ± S.D.

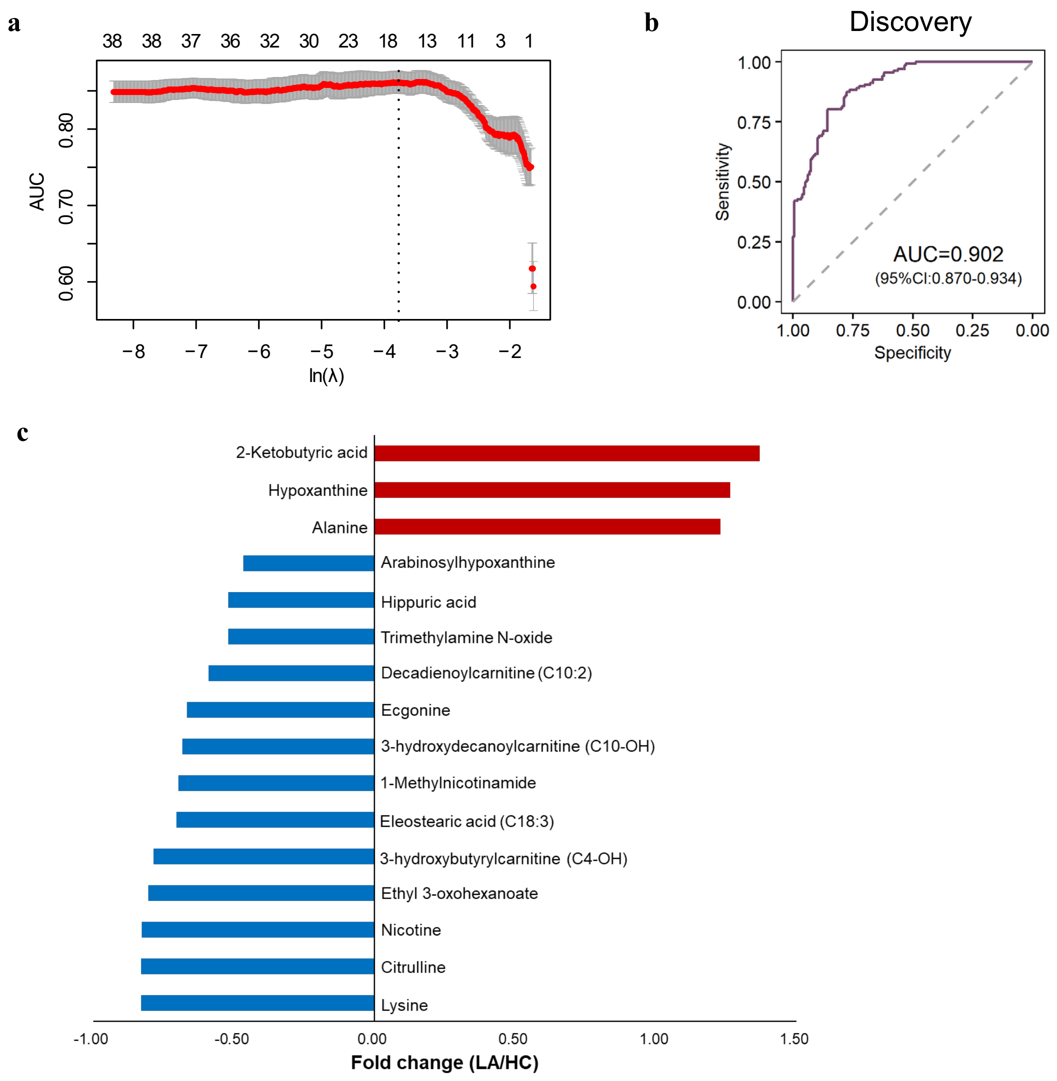

Supplementary Figure 6. Construction of classification model for discrimination between lung adenocarcinoma and healthy subjects. a, Cross-validation statistics of LASSO regression model for selection of metabolic biomarkers. Numbers above indicates the average number of selected biomarkers under a given λ. The red dotted line indicates the mean values of AUC under the corresponding λ. The grey error band indicates the minimum and maximum values of AUC. The dashed line points to the optimal model with 16 selected biomarkers. **b**, The areas under the receiver operating characteristic (ROC) curve (AUC) showing the efficacy of the discriminant model based on the combination of 16 metabolites. **c**, Fold changes of 16 selected metabolites in LA group compared with HC group. LA, n=136; HC, n=174.

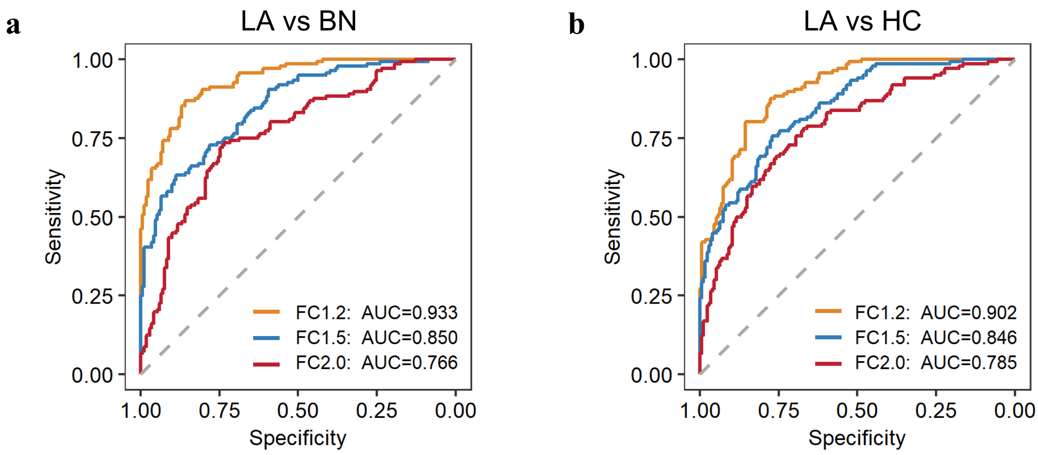

Supplementary Figure 7. Comparison of AUC values based on different fold change cutoffs of differential metabolites between two groups in the discovery cohort. a, AUC values of the metabolic classifier model to distinguish between lung adenocarcinoma (LA) and benign nodules (BN). **b**, AUC values of the metabolic classifier model to distinguish between lung adenocarcinoma (LA) and healthy controls (HC). FC 1.2 (orange line), fold change=1.2; FC1.5 (blue line), fold change=1.5; FC 2.0 (red line), fold change =2.

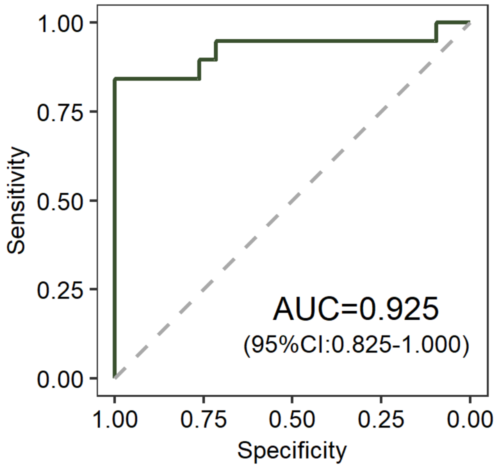

Supplementary Figure 8. **External validation from an outside lab for discriminating between benign and malignant pulmonary nodules.** The areas under receiver operating characteristic (ROC) curve (AUC) showing the diagnostic capacity based on the established metabolic classifier. LA, n=19; BN, n=21.

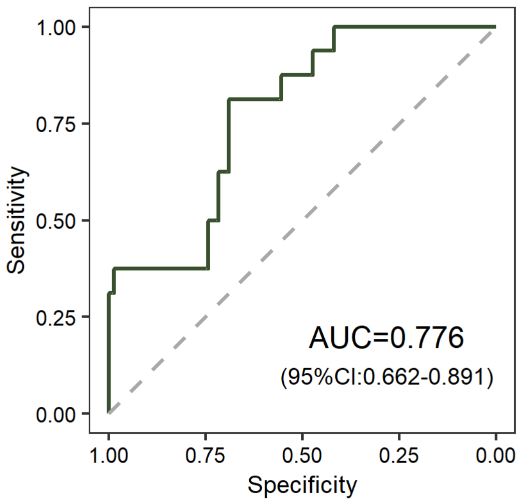

Supplementary Figure 9. Estimation of the metabolic classifier for discrimination between lung squamous cell carcinoma (LUSC) and benign pulmonary nodule (BN). The areas under receiver operating characteristic (ROC) curve (AUC) showing the diagnostic capacity based on the established metabolic classifier. LUSC, n=16; BN, n=74.

a

b

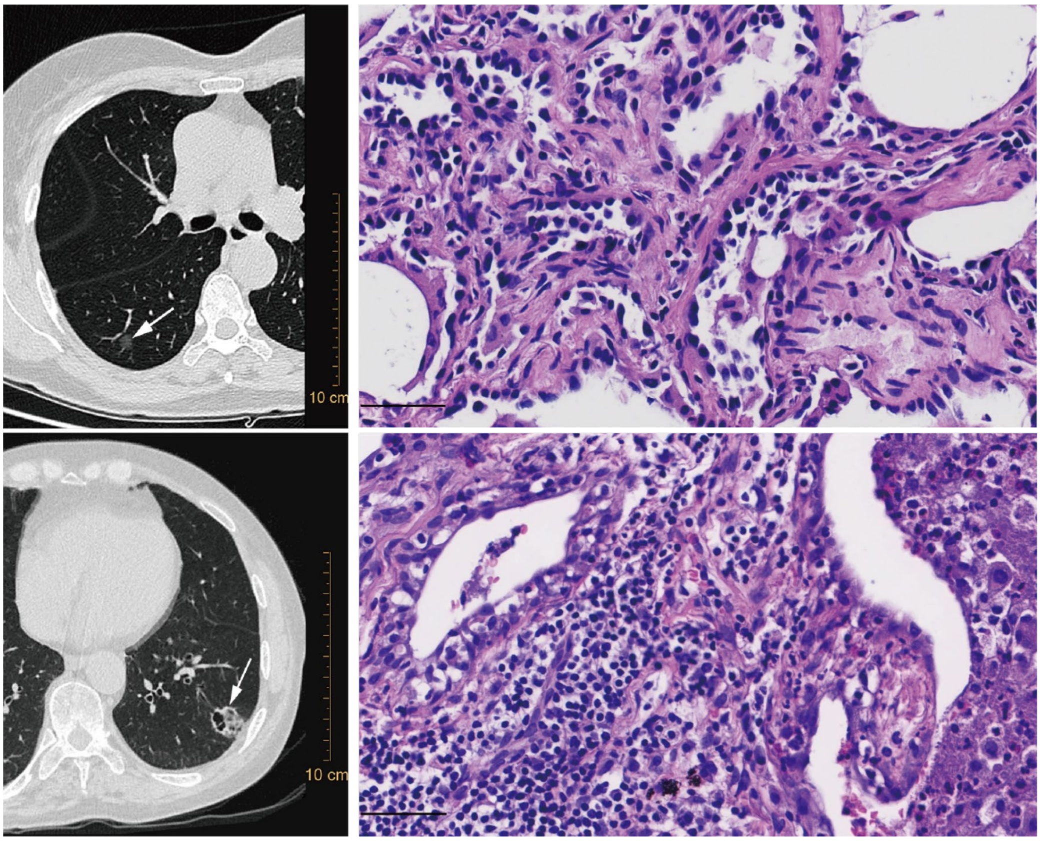

Supplementary Figure 10. Two representative cases of indeterminate pulmonary nodules (IPN) by CT evaluation in the external validation cohort. a, The CT image on axial lung window view showing an 8 mm nodule with irregular margin (arrow). The sample was predicted as a malignant nodule according to the cutoff value of the metabolic classifier. H&E staining of lung tissue from resection surgery indicated minimally invasive adenocarcinoma. **b,** The CT image on axial lung window view showing a 23 mm subpleural cavitated nodule. The sample was predicted as a benign nodule according to the cutoff value of the metabolic classifier. H&E staining of lung tissue from resection surgery showed findings consistent with chronic bronchitis. No signs of malignancy were detected by histopathological examination. Scale bar=50 μm. The H&E staining images are representative views of multiple (>3) microscopic fields examined by the pathologist.

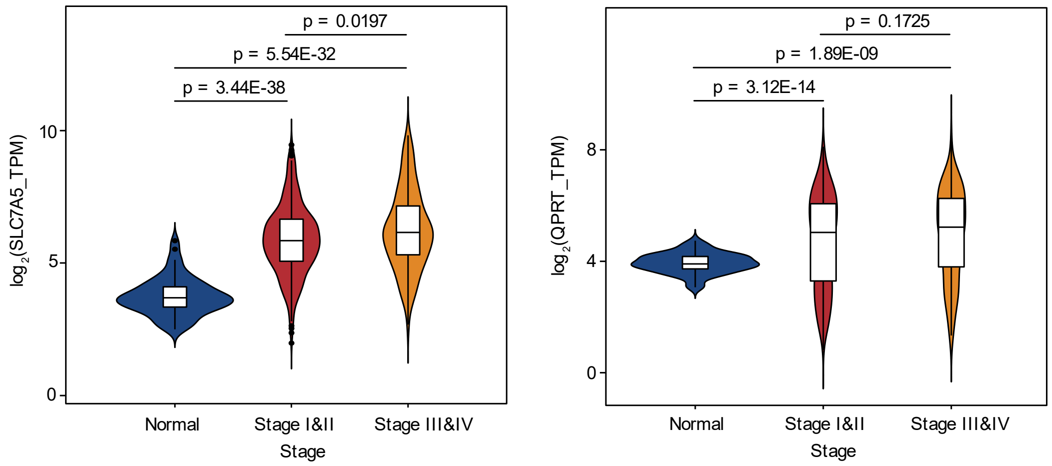

Supplementary Figure 11. Normalized Log_2TPM (transcript per million) mRNA expression of SLC7A5 and QPRT in lung adenocarcinoma vs normal lung tissues in the LUAD-TCGA dataset. Normal, n=58; Stage I&II, n=395; Stage III&IV, n=110. The white box represents the interquartile range, the horizontal black line in the center indicates the median and the vertical black line extended from the box indicates 95% confidence intervals (CI). Two-sided Student's t-test was used.

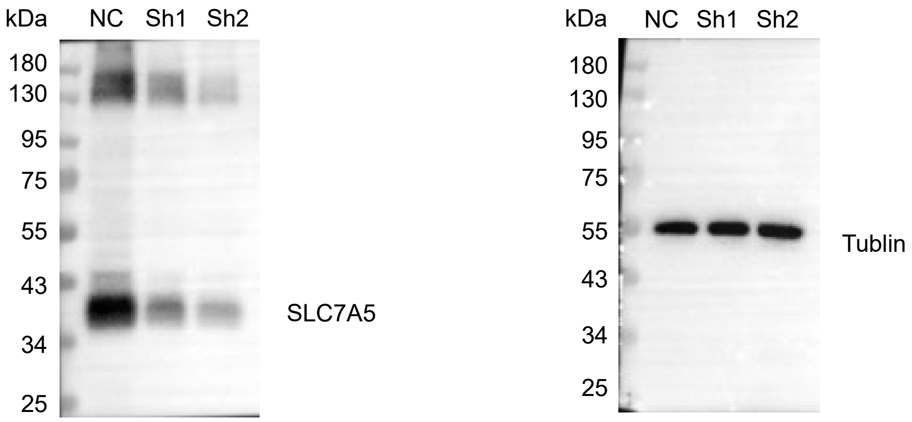

Supplementary Figure 12. Knockdown of SLC7A5 in A549 cells by shRNA. Westernblot analysis showing protein expression of SLC7A5 (molecular weight, 39 kDa) and Tublin (molecular weight, 55 kDa). NC, shRNA-nonspecific control. Sh1&2, shSLC7A5 sequence 1 and 2. Numbers on the left indicate molecular weights of corresponding markers. The experiment was performed three times independently with similar results.

Supplementary Table 1. Demographic characteristics of samples for global metabolomic study.

	Discovery			Internal validation		External validation	
	Healthy control	Benign nodule	Stage I lung adenocarcinoma	Benign nodule	Stage I lung adenocarcinoma	Benign nodule	Stage I lung adenocarcinoma
Subjects, N	174	170	136	74	30	48	63
Gender, N (Male/Female)	87/87	85/85	65/71	37/37	15/15	30/18	26/37
Age, Mean ± SD, years	55±9	54±8	56±10	54±8	56±10	48±10	58±8
Smoking status, N (%)							
Current	29 (17%)	26 (15%)	25 (18%)	13 (18%)	4 (13%)	9 (19%)	7 (11%)
Quit	7 (4%)	10 (6%)	12 (9%)	3 (4%)	3 (10%)	3 (6%)	10 (16%)
Never	135 (78%)	133 (78%)	98 (72%)	58 (78%)	23 (77%)	36 (75%)	45 (71%)
Unknown	3 (2%)	1 (1%)	1 (1%)	/	/	/	1 (2%)
Tumor/Nodule size, maximal diameter (mm)	/	2~19	6~30	3~19	7~30	5~23	6~30

Supplementary Table 2. Annotated differential metabolites in serum of lung adenocarcinoma compared with benign nodules and healthy controls.

No.	HMDB ID	Name	Class	LA vs HC		LA vs BN		BN vs HC	
				FC	FDR	FC	FDR	FC	FDR
1	HMDB0013325	Decadienoylcarnitine (C10:2)	Acyl carnitine	0.59	1.07E-13	0.73	3.42E-05	0.80	4.46E-04
2	HMDB0000791	Octanoylcarnitine (C8)	Acyl carnitine	0.62	3.97E-10	0.75	4.45E-06	/	/
3	HMDB0000651	Decanoylcarnitine (C10)	Acyl carnitine	0.62	2.03E-09	0.74	7.39E-06	/	/
4	HMDB0013324	Octenoylcarnitine (C8:1)	Acyl carnitine	0.69	3.22E-05	0.69	1.27E-05	/	/
5	HMDB0000756	Hexanoylcarnitine (C6)	Acyl carnitine	0.69	2.95E-07	0.83	4.20E-04	/	/
6	HMDB0061638	3-hydroxydodecanoylcarnitine (C12-OH)	Acyl carnitine	0.62	3.60E-11	0.61	2.21E-07	/	/
7	HMDB0061636	3-hydroxydecanoylcarnitine (C10-OH)	Acyl carnitine	0.68	5.10E-12	0.66	2.33E-09	/	/
8	HMDB0061634	3-hydroxyoctanoylcarnitine (C8-OH)	Acyl carnitine	0.72	5.67E-10	0.69	4.54E-09	/	/
9	HMDB0006548	Ecgonine	Alkaloid	0.67	1.92E-09	0.67	1.76E-08	/	/
10	HMDB0000925	Trimethylamine N-oxide	Amine	0.52	4.92E-09	0.69	4.31E-06	/	/
11	HMDB0000699	1-Methylnicotinamide	Amine	0.70	3.46E-10	0.67	1.42E-07	/	/
12	HMDB0001885	3-Chlorotyrosine	Amino acid	0.46	1.09E-08	0.60	2.86E-06	/	/
13	HMDB0000670	Homo-L-arginine	Amino acid	0.74	1.18E-07	0.76	1.67E-06	/	/
14	HMDB0000259	Serotonin	Amino acid	0.81	3.00E-04	0.83	9.06E-04	/	/
15	HMDB0000161	Alanine	Amino acid	1.23	8.28E-06	1.24	6.18E-06	/	/
16	HMDB0030963	Eleostearic acid (C18:3)	Fatty acid	0.70	2.52E-09	0.71	1.10E-06	/	/
17	HMDB0031307	Ethyl 3-oxohexanoate	Fatty acid	0.80	1.37E-05	0.78	2.54E-05	/	/
18	HMDB0000195	Inosine	Nucleoside	0.41	4.34E-12	0.38	4.43E-12	/	/
19	HMDB0003040	Arabinosylhypoxanthine	Nucleoside	0.47	5.51E-11	0.41	4.34E-11	/	/
20	HMDB0000714	Hippuric acid	Organic acid	0.52	9.60E-09	0.64	1.90E-06	/	/
21	HMDB0031403	Cyclohexaneacetic acid	Organic acid	0.76	2.38E-03	0.80	7.51E-05	/	/
22	HMDB0000190	Lactic acid	Organic acid	1.24	2.23E-07	1.30	3.59E-10	/	/
23	HMDB0000005	2-Ketobutyric acid	Organic acid	1.37	9.01E-04	1.35	2.18E-03	/	/
24	HMDB0000243	Pyruvic acid	Organic acid	1.44	7.14E-08	1.39	3.43E-06	/	/
25	HMDB0000157	Hypoxanthine	Purine	1.27	1.82E-09	1.28	1.85E-09	/	/
26	HMDB0000292	Xanthine	Purine	1.28	3.08E-07	1.28	2.74E-06	/	/
27	HMDB0001325	N6,N6,N6-Trimethylysine	Amino acid	0.81	2.73E-04	/	/	0.70	1.33E-07
28	HMDB0000684	Kynurenine	Amino acid	/	/	0.78	4.81E-05	1.25	2.08E-03
29	HMDB0002014	Tetradecenoylcarnitine (C14:1)	Acyl carnitine	/	/	0.77	5.47E-04	/	/
30	HMDB0002183	Docosahexaenoic acid (C22:6)	Fatty acid	/	/	0.78	1.54E-05	/	/
31	HMDB0250194	Choline Sulfate	Organic acid	/	/	0.72	1.08E-06	/	/
32	HMDB0000079	Dihydrothymine	Pyrimidine	/	/	1.30	1.63E-03	/	/
33	HMDB0000416	17-Hydroxypregnenolone sulfate	Sulfated steroid	/	/	1.22	6.77E-03	/	/
34	HMDB0000774	Pregnenolone sulfate	Sulfated steroid	/	/	1.47	2.98E-02	/	/
35	HMDB0002366	Tiglylcarnitine (C5:1)	Acyl carnitine	0.82	1.56E-04	/	/	/	/
36	HMDB0000824	Propionylcarnitine (C3)	Acyl carnitine	0.82	3.99E-07	/	/	/	/
37	HMDB0062735	3-hydroxybutyrylcarnitine (C4-OH)	Acyl carnitine	0.78	2.16E-04	/	/	/	/
38	HMDB0061918	Oxindole	Alkaloid	0.80	1.97E-03	/	/	/	/
39	HMDB0001934	Nicotine	Alkaloid	0.83	9.43E-03	/	/	/	/
40	HMDB0003045	Ergothioneine	Amino acid	0.79	9.12E-04	/	/	/	/
41	HMDB0006344	Phenylacetylglutamine	Amino acid	0.80	2.65E-02	/	/	/	/
42	HMDB0000904	Citrulline	Amino acid	0.83	3.53E-08	/	/	/	/
43	HMDB0000182	Lysine	Amino acid	0.83	6.90E-10	/	/	/	/
44	HMDB0001901	Aminocaproic acid	Fatty acid	1.29	4.70E-03	/	/	/	/
45	HMDB0002820	Methylimidazoleacetic acid	Organic acid	0.82	1.85E-02	/	/	/	/

LA, lung adenocarcinoma; BN, benign nodules; HC, healthy controls. FC, fold change.

Supplementary Table 3. Estimated weights of the 27 selected metabolites (M1-27) in the logistic regression model for distinguishing lung adenocarcinoma and benign nodules.

No.	Metabolites	Weights
M_1	Hypoxanthine	2.29
M_2	Alanine	1.02
M_3	2-Ketobutyric acid	0.64
M_4	Decadienoylcarnitine (C10:2)	0.62
M_5	Xanthine	0.47
M_6	17-Hydroxypregnenolone sulfate	0.42
M_7	Dihydrothymine	0.38
M_8	Octanoylcarnitine (C8)	0.26
M_9	Lactic acid	0.05
M_{10}	Pregnenolone sulfate	0.03
M_{11}	3-Chlorotyrosine	-0.05
M_{12}	Cyclohexaneacetic acid	-0.12
M_{13}	Choline Sulfate	-0.16
M_{14}	Trimethylamine N-oxide	-0.17
M_{15}	Octenoylcarnitine (C8:1)	-0.36
M_{16}	1-Methylnicotinamide	-0.40
M_{17}	Serotonin	-0.45
M_{18}	Docosahexaenoic acid (C22:6)	-0.46
M_{19}	Decanoylcarnitine (C10)	-0.47
M_{20}	Eleostearic acid (C18:3)	-0.53
M_{21}	Homo-L-arginine	-0.55
M_{22}	Pyruvic acid	-0.79
M_{23}	3-hydroxydecanoylcarnitine (C10-OH)	-0.95
M_{24}	Ecgonine	-1.02
M_{25}	Kynurenine	-1.19
M_{26}	Ethyl 3-oxohexanoate	-1.52
M_{27}	Arabinosylhypoxanthine	-1.88
	constant	4.01

The probability (P) value of each sample is calculated with levels of the corresponding metabolites as the following formula:

$\ln[P/(1-P)] = 2.29\times M_1 + 1.02\times M_2 + 0.64\times M_3 + 0.62\times M_4 + 0.47\times M_5 + 0.42\times M_6 + 0.38\times M_7 + 0.26\times M_8 + 0.05\times M_9 + 0.03\times M_{10} - 0.05\times M_{11} - 0.12\times M_{12} - 0.16\times M_{13} - 0.17\times M_{14} - 0.36\times M_{15} - 0.4\times M_{16} - 0.45\times M_{17} - 0.46\times M_{18} - 0.47\times M_{19} - 0.53\times M_{20} - 0.55\times M_{21} - 0.79\times M_{22} - 0.95\times M_{23} - 1.02\times M_{24} - 1.19\times M_{25} - 1.52\times M_{26} - 1.88\times M_{27} + 4.01$

Supplementary Table 4. Demographic characteristics of the samples for targeted metabolomic study.

Characteristics	Healthy control	Benign nodule	Stage I lung adenocarcinoma
Subjects, N	70	70	70
Gender, N (Male/Female)	32/38	37/33	37/33
Age, Mean ± SD, years	56±9	54±8	57±9
Smoking status, N (%)			
Current	8 (11%)	13 (19%)	14 (20%)
Quit	3 (4%)	5 (7%)	9 (13%)
Never	56 (80%)	51 (73%)	46 (66%)
Unknown	3 (4%)	1 (1%)	1 (1%)

Supplementary Table 5. Demographic characteristics of the lung squamous cell carcinoma (LUSC) samples and benign nodules from the internal validation set.

Characteristics	Benign nodule	Stage I lung squamous cell carcinoma
Subjects, N	74	16
Gender, N (Male/Female)	37/37	15/1
Age, Mean ± SD, years	54±8	65±6
Smoking status, N (%)		
Current	13 (18%)	6 (38%)
Quit	3 (4%)	7 (44%)
Never	58 (78%)	3 (19%)

Supplementary Table 6. RSD values of internal standards before and after normalization by reference serum.

	Sample		QC	
	raw data	corrected data	raw data	corrected data
$^{13}C_6$-Ile	44.8%	7.9%	43.4%	7.1%
^{13}C-Met	42.0%	7.9%	44.0%	7.4%
$^{13}C_3$-Pyr	16.3%	10.6%	15.1%	5.5%
^{13}C-Lac	16.9%	9.9%	14.6%	8.2%

Relative standard deviation of absolute abundance (raw data) and relative abundance (corrected data) of 4 internal standards in experimental samples and QC samples from the discovery set. $^{13}C_6$-isoleucine ($^{13}C_6$-Ile) and ^{13}C-methionine (^{13}C-Met) were extracted in +ESI mode, $^{13}C_3$-pyruvate ($^{13}C_3$-Pyr) and ^{13}C-lactate(^{13}C-Lac) were extracted in -ESI mode.
