# Supplementary material for: Metabolomic differentiation of benign vs malignant pulmonary nodules with high specificity via high-resolution mass spectrometry analysis of patient sera
Source: Nat Commun. 2023 Apr 24;14:2339. doi: 10.1038/s41467-023-37875-1 (PMC10126054; doi:10.1038/s41467-023-37875-1)
Supplement: Supplementary file 2 — Reporting Summary [file 41467_2023_37875_MOESM2_ESM.pdf]

nature portfolio

Corresponding author(s): Yumin Hu,Wanli Liu, Tiangang Luan

Last updated by author(s): Mar 2, 2023

Reporting Summary

Nature Portfolio wishes to improve the reproducibility of the work that we publish. This form provides structure for consistency and transparency in reporting. For further information on Nature Portfolio policies, see our Editorial Policies and the Editorial Policy Checklist.

Statistics

For all statistical analyses, confirm that the following items are present in the figure legend, table legend, main text, or Methods section.

n/a	Confirmed	
[ ]	[x]	The exact sample size (n) for each experimental group/condition, given as a discrete number and unit of measurement
[ ]	[x]	A statement on whether measurements were taken from distinct samples or whether the same sample was measured repeatedly
[ ]	[x]	The statistical test(s) used AND whether they are one- or two-sided<br>*Only common tests should be described solely by name; describe more complex techniques in the Methods section.*
[x]	[ ]	A description of all covariates tested
[ ]	[x]	A description of any assumptions or corrections, such as tests of normality and adjustment for multiple comparisons
[ ]	[x]	A full description of the statistical parameters including central tendency (e.g. means) or other basic estimates (e.g. regression coefficient) AND variation (e.g. standard deviation) or associated estimates of uncertainty (e.g. confidence intervals)
[ ]	[x]	For null hypothesis testing, the test statistic (e.g. F, t, r) with confidence intervals, effect sizes, degrees of freedom and P value noted<br>*Give P values as exact values whenever suitable.*
[x]	[ ]	For Bayesian analysis, information on the choice of priors and Markov chain Monte Carlo settings
[x]	[ ]	For hierarchical and complex designs, identification of the appropriate level for tests and full reporting of outcomes
[ ]	[x]	Estimates of effect sizes (e.g. Cohen's d, Pearson's r), indicating how they were calculated

Our web collection on statistics for biologists contains articles on many of the points above.

Software and code

Policy information about availability of computer code

Data collection	Untargeted metabolomics data of LC-MS was acquired by Xcalibur 4.1 softwarew (Thermo Fisher Scientific) . Targeted metabolomics data of LC-MS was collected by Mass Hunter B.07.00 (Agilent Technologies).
Data analysis	Metabolomic analysis was performed by MCompound Discovery 3.1 and TraceFinder 4.0 (Thermo Fisher Scientific).Fold changes, Wilcoxon rank-sum test and KEGG pathway analysis were analyzed by MetaboAnalyst 5.0 (https://www.metaboanalyst.ca). PCA and PLS-DA analysis were analyzed by ropls (v.1.26.4) R package. LASSO binary logistic regression was analyzed by glmnet (v.4.1-3) R package. ROC analysis was performed by pROC (v.1.18.0.) R package. Heatmap was performed by pheatmap (v1.0.12) R package.

For manuscripts utilizing custom algorithms or software that are central to the research but not yet described in published literature, software must be made available to editors and reviewers. We strongly encourage code deposition in a community repository (e.g. GitHub). See the Nature Portfolio guidelines for submitting code & software for further information.

Data

Policy information about availability of data

All manuscripts must include a data availability statement. This statement should provide the following information, where applicable:

- Accession codes, unique identifiers, or web links for publicly available datasets
- A description of any restrictions on data availability
- For clinical datasets or third party data, please ensure that the statement adheres to our policy

The raw MS data of extracted features and the normalized MS data by reference serum are shown in Supplementary data 1 and Supplementary data 2, respectively.

Peak annotation of differential features is provided in Supplementary data 3. The LUAD dataset from TCGA can be downloaded from https://portal.gdc.cancer.gov/. Raw data of plotting figures are provided in Source Data. All source data are provided with this paper.

Human research participants

Policy information about studies involving human research participants and Sex and Gender in Research.

Reporting on sex and gender	Gender information was collected based on self-reporting and informed consent was obtained by all participants. To minimize gender bias, an approximately equal number of male and female cases were assigned to each group (healthy, benign, adenocarcinoma) of the discovery and internal validation cohorts. However, as the aim of our study was to establish a classifier to distinguish between early-stage lung cancer and benign adenocarcinoma in a general population, gender/sex was not included as a parameter for model training. The model also achieved a desirable AUC in both internal and external validation cohorts, indicating the applicability of the model in a general population.
Population characteristics	A total of 480 serum samples collected from Sun Yat-sen University Cancer Center, including 174 healthy controls (HC), 170 benign nodules (BN) and 136 stage I lung adenocarcinoma (LA) were used as the discovery cohort. 104 samples collected from Sun Yat-sen University Cancer Center and 111 samples collected from another two hospitals were assigned for internal and external validation, respectively. 16 cases of stage I lung squamous cell carcinoma were also collected from Sun Yat-sen University Cancer Center. Detailed characteristics are provided in Supplementary Table 1, 4, 5 in the indicated experiments.
Recruitment	Samples for the discovery and internal validation cohorts were collected between January 2018 and May 2020 from Sun Yat-sen University Cancer Center. Samples for the external validation cohorts were collected between August 2021 to October 2022 from The Affiliated Cancer Hospital of Zhengzhou University and The First Affiliated Hospital of Sun Yat-sen University. Informed consents were obtained from all participants and no compensation was provided.
Ethics oversight	The study was approved by the Ethics Committee of Sun Yat-Sen University Cancer Center, The First Affiliated Hospital of Sun Yat-sen University, and the Affiliated Cancer Hospital of Zhengzhou University.

Note that full information on the approval of the study protocol must also be provided in the manuscript.

Field-specific reporting

Please select the one below that is the best fit for your research. If you are not sure, read the appropriate sections before making your selection.

[x] Life sciences [ ] Behavioural & social sciences [ ] Ecological, evolutionary & environmental sciences

For a reference copy of the document with all sections, see nature.com/documents/nr-reporting-summary-flat.pdf

Life sciences study design

All studies must disclose on these points even when the disclosure is negative.

Sample size	No statistical method was used to pre-determine sample size of patient samples. Previous metabolomic studies (PMID: 29437793; PMID: 32678093) for biomarker discovery were considered as a reference for size determination and our samples were adequate compared with these reports.
Data exclusions	No data is excluded in the current study.
Replication	Three independent assays were performed for measurement of cellular levels of tryptophan, NADt and glycolytic activity in A549 cells with similar results. Knockdown of SLC7A5 was verified three times by western-blot analysis. The number of biological replications are as indicated in figure legends and methods with successful attempts. The reproducibility of serum metabolomics data was based on the strict quality control (See methods, Supplementary Figure 2 and Supplementary Table 6).
Randomization	Serum samples were randomly allocated to the discovery (306, 74.6%) and the internal validation cohorts (104, 25.4%). Samples in each batch were tested on the UPLC-HRMS platform in random order. For targeted metabolomic study, 70 cases in each group were randomly selected from the discovery set.
Blinding	The investigators were blinded to group allocation when performed LC-MS data acquisition and data analysis.

Reporting for specific materials, systems and methods

We require information from authors about some types of materials, experimental systems and methods used in many studies. Here, indicate whether each material, system or method listed is relevant to your study. If you are not sure if a list item applies to your research, read the appropriate section before selecting a response.

Materials & experimental systems

n/a	Involved in the study	
☐	☒	Antibodies
☐	☒	Eukaryotic cell lines
☒	☐	Palaeontology and archaeology
☒	☐	Animals and other organisms
☒	☐	Clinical data
☒	☐	Dual use research of concern

Methods

n/a	Involved in the study	
☒	☐	ChIP-seq
☒	☐	Flow cytometry
☒	☐	MRI-based neuroimaging

Antibodies

Antibodies used	SLC7A5 (Cell Signaling Technology, 5347); Tublin, (Cell Signaling Technology, 2148).The antibody dilution of SLC7A5 and tublin is 1:1000 for westernblot analysis.
Validation	SLC7A5 (also known as LAT1):<br>https://www.cellsignal.com/products/primary-antibodies/lat1-antibody/5347; The link contains a western blot analysis image showing extracts from HT-1080 and MCF7 cells using LAT 1 antibody; Species reactivity: human; 52 product citation including Elife, Cell Reports, PLoS Pathology are also shown for western blot application .<br><br>Tublin:<br>https://www.cellsignal.com/products/primary-antibodies/a-b-tubulin-antibody/2148; The link contains a western blot analysis image showing extracts from HeLa, NIH/3T3, C6 and COS-7 cells, using α/β-Tubulin Antibody; Species reactivity: human;640 product citation are shown.

Eukaryotic cell lines

Policy information about cell lines and Sex and Gender in Research

Cell line source(s)	The A549 cell line is from American Type Culture Collection.
Authentication	Authentication was performed by STR profiling.
Mycoplasma contamination	The cell line used in this study was tested and negative for mycoplasma contamination.
Commonly misidentified lines (See ICLAC register)	None.
