# Supplementary material for: Metabolomic differentiation of benign vs malignant pulmonary nodules with high specificity via high-resolution mass spectrometry analysis of patient sera
Source: Nat Commun. 2023 Apr 24;14:2339. doi: 10.1038/s41467-023-37875-1 (PMC10126054; doi:10.1038/s41467-023-37875-1)
Supplement: Supplementary file 3 — Description of Additional Supplementary Files [file 41467_2023_37875_MOESM3_ESM.docx]

**Description of Additional Supplementary Files**

Supplementary Data 1

Description: Raw metabolomics mass spectrometry data.

Supplementary Data 2

Description: Corrected metabolomics data.

Supplementary Data 3

Description: Identification results of differential features.
